# Evaluating clinical impact of a shortened infusion duration for ramucirumab: a model-based approach

**DOI:** 10.1007/s00280-020-04223-9

**Published:** 2021-02-02

**Authors:** Ling Gao, Yiu-Keung Lau, Ran Wei, Lisa O’Brien, Amanda Long, Yongzhe Piao, Paolo Abada

**Affiliations:** 1 Eli Lilly and Company, Lilly Corporate Center, Indianapolis, IN 46285 USA; 2 Eli Lilly Japan K.K., Kobe, Japan

**Keywords:** Ramucirumab, Infusion-related reactions, Infusion duration, Pharmacokinetics, Vascular endothelial growth factor receptor-2 antagonist

## Abstract

**Purpose:**

We investigated the impact of infusion duration (30 and 60 min) on the pharmacokinetic profile of ramucirumab using a population pharmacokinetic (PopPK) modeling approach. We also assessed the relationship between infusion rate and incidence of immediate infusion-related reactions (IRRs; occurring on the day of administration) using ramucirumab phase II/III study data.

**Methods:**

The impact of different infusion durations (30 vs. 60 min) on the time-course of ramucirumab concentration profiles were evaluated using a PopPK model, established using ramucirumab pharmacokinetic data from 2522 patients. Logistic regression was used to evaluate the association between ramucirumab infusion rate and incidence of immediate IRRs in clinical trials.

**Results:**

Ramucirumab time-course concentration profiles were equivalent following a 30- or 60-min infusion. In the pooled clinical study dataset, 254 of 3216 (7.9%) patients receiving ramucirumab experienced at least one immediate IRR (any grade). When grouped according to infusion rate quartile, the incidence of immediate IRRs (any grade or grade ≥ 3) was similar across quartiles; findings were confirmed in sensitivity analyses. The risk of immediate IRRs was not found to be associated with infusion rate based on multivariate logistic analysis.

**Conclusion:**

Shortening the infusion duration of ramucirumab from 60 to 30 min has no impact on ramucirumab exposure. Analysis of trial data found no relationship between an increased risk of immediate IRRs and a faster infusion rate. Such a change in infusion duration is unlikely to affect the clinical efficacy or overall safety profile of ramucirumab.

**Supplementary Information:**

The online version contains supplementary material available at 10.1007/s00280-020-04223-9.

## Introduction

Lengthy intravenous infusions are burdensome and inconvenient for patients. Patients with cancer have indicated a strong preference for receiving drugs with shorter infusion times as they are less disruptive to their lives [1]. Infusions are usually administered in an outpatient setting, and long infusion times necessitate lengthy observation periods with increased nursing and administration staff workloads. Shorter infusion times have been associated with notable reductions in healthcare resources, medical personnel time [2, 3], and time in care; potentially allowing more patients to be treated in a day [1].

Ramucirumab is a human recombinant immunoglobin (Ig)G1 monoclonal antibody (mAb) antagonist of vascular endothelial growth factor receptor-2 currently approved for the second-line treatment of advanced or metastatic gastric or gastroesophageal junction adenocarcinoma, metastatic non-small-cell lung cancer [in combination with docetaxel or erlotinib in patients with epidermal growth factor receptor exon 19 deletions or exon 21 (L858R) mutations], metastatic colorectal cancer, and hepatocellular carcinoma with elevated alpha fetoprotein levels. The recommended initial dosing regimen for ramucirumab, either as monotherapy or in combination with chemotherapy, is 8 mg/kg or 10 mg/kg every 2 weeks (Q2W) or 10 mg/kg every 3 weeks (Q3W) administered as an intravenous infusion over 60 min following premedication with a histamine-1 receptor antagonist [4].

The efficacy and overall safety of antibody therapies such as ramucirumab have been linked to exposure to the drug [5, 6]. Hence, while a shortened ramucirumab infusion duration could benefit both patients and healthcare staff, it is also important to consider the impact such a change may have on the pharmacokinetic (PK) profile of the drug. Population PK (PopPK) modeling is widely used during drug development to describe and predict the concentration profile of a drug over time [7, 8]. The pharmacokinetics of ramucirumab have previously been well-described by a linear two-compartment pharmacostatistical model which incorporated the effect of body weight on ramucirumab clearance and central compartment volume [9]. Other patient-related factors such as age, sex, race, cancer type, various measures of liver and renal function as well as baseline measures of tumor burden were not found to be statistically and/or clinically relevant factors in the disposition of ramucirumab [9].

PopPK modeling was applied to predict exposure parameters for individual patients for use in determining PK/pharmacodynamic relationships for ramucirumab from both safety and efficacy perspectives [5, 6, 10].

A faster infusion rate is also perceived as a potential risk factor for infusion-related reactions (IRRs), common adverse events associated with mAb infusions [11]. In patients who have previously experienced a grade 1–2 IRR, standard clinical practice is to reduce the infusion rate during drug rechallenge [11]. However, the relationship between infusion rate and IRR incidence is not well defined.

We developed a PopPK model to simulate and compare ramucirumab PK profiles following either a 30- or 60-min infusion at the two approved dosing regimens to evaluate the potential impact of a change in infusion duration on the clinical efficacy and overall safety profile of the drug. Additionally, the relationship between ramucirumab infusion rate and the incidence of immediate IRRs was investigated using data from key phase II and III studies of ramucirumab.

## Materials and methods

### PopPK model development and simulation

Emerging evidence has suggested that monoclonal antibodies may exhibit time-dependent clearance (CL) due to changes in oncologic disease status [12–14]. In this updated PopPK analysis, temporal changes in CL were incorporated into the two-compartment linear model developed by O’Brien et al. [9] with a sigmoidal function as described for nivolumab [12]:

$$CL = CL_{0} \cdot exp\left( {\frac{{E_{{max}} \cdot Time^{{C^{\prime\prime\prime}}} }}{{T_{{50}}^{{C^{\prime\prime\prime}}} + Time^{{C^{\prime\prime\prime}}} }}} \right)$$

where CL_0_ represents clearance (CL) at time of first dose administration, E_max_ represents maximal change in CL, T_50_ is the time at which the change in CL is 50% of E_max_, and ƴ represents the sigmoidicity of the relationship with time.

The effect of body weight on both CL and central volume of distribution (V_1_) was included in the base pharmacostatistical model. Additional patient-related factors including age, sex, race, Eastern Cooperative Oncology Group (ECOG) performance status, cancer type, and various measures of liver and renal function were also assessed. Covariate evaluation and final model development were performed as described in O’Brien [9]. In addition to standard goodness-of-fit plots, model evaluation also included a visual predictive check (VPC) to assess the general predictability of the model by visually examining the observed versus predicted profiles. The distribution (median, 5th, and 95th percentiles) of simulated profiles was overlaid on observed data. Plotted data were stratified by duration of treatment (< 27 days and ≥ 27 days), corresponding to estimated T_50_, to assess model fit of early timepoints and later timepoints.

The model was developed using concentration–time data (for pharmacokinetic parameters see online Supplementary Material, Table S1) collected from 2522 patients in 17 clinical studies (data on file, Eli Lilly and Company; see online Supplementary Material, Table S2). All patients provided written informed consent and the trials were designed and conducted to follow Good Clinical Practice guidelines and in accordance with the Declaration of Helsinki. Ramucirumab was administered as an intravenous infusion over approximately 60 min. Patients included in the pooled analysis received one of five treatment regimens: ramucirumab 8 mg/kg every 2 weeks (Q2W), 12 mg/kg Q2W, 6 mg/kg once weekly (QW), 8 mg/kg on days 1 and 8 every 3 weeks (Q3W), or 10 mg/kg Q3W.

The final PK model was used to simulate concentration–time profiles and exposure parameters following a 30- or 60-min infusion with ramucirumab 8 mg/kg Q2W or 10 mg/kg Q3W. The simulation dataset included 500 patients for each infusion–regimen combination. Dose amounts were determined using 500 weights randomly sampled without replacement from the baseline weights of the 2522 patients included in the PopPK analysis. Modeling and simulations were performed using NONMEM 7.4.2 [15].

### Immediate IRRs

Clinical data from all eight phase III trials evaluating commercial formulations and two phase II studies evaluating ramucirumab 12 mg/kg (Table 1) were included in analyses to evaluate the association between ramucirumab infusion rate and the incidence of immediate IRRs (IRRs occurring on the day of ramucirumab infusion). All included trials required premedication. As dosing of ramucirumab was weight based, the total amount of ramucirumab administered varied for each patient, making it possible to observe different ramucirumab infusion rates. While the recommended infusion time was approximately 60 min, several studies allowed some flexibility in initial infusion time (± ≤ 15 min), and infusions could be longer if needed to maintain an infusion rate of ≤ 25 mg/min. Sensitivity analyses were therefore performed using infusion rates derived from a standard 60-min infusion. (For details of the methodology used to identify immediate IRRs, calculate immediate IRR incidence, and investigate the relationship between immediate IRR incidence and infusion rate, see the online Supplementary Material.)

**Table 1:** Summary of immediate infusion-related reactions in ramucirumab-treated patients by study and overall

Study^a^	Phase	Indication	RAM dosing regimen	Patients with ≥ 1 immediate IRR (narrow terms^b^)	
				n/*N*	%
REGARD [21] NCT00917384	III	Second-line GC	8 mg/kg Q2W	6/236	2.5
RAINBOW^c^ [22] NCT01170663	III	Second-line GC	8 mg/kg Q2W	45/327	13.8
REVEL^c^ [23] NCT01168973	III	Second-line NSCLC	10 mg/kg Q3W	51/626	8.1
RAISE^c^ [24] NCT01183780	III	Second-line CRC	8 mg/kg Q2W	46/528	8.7
REACH [25] NCT01140347	III	Second-line HCC	8 mg/kg Q2W	41/317	12.9
REACH-2 [26] NCT02435433	III	Second-line HCC	8 mg/kg Q2W	17/197	8.6
RAINFALL^c^ [27] NCT02314117	III	First-line GC	8 mg/kg D1D8 Q3W	15/323	4.6
RANGE^c^ [28] NCT02426125	III	Second-line UC	10 mg/kg Q3W	18/258	7.0
NCT02443883 (I4T-MC-JVDB)	II	Second-line GC	8 mg/kg Q2W 12 mg/kg Q2W 6 mg/kg QW 8 mg/kg D1D8 Q3W	4/161	2.5
NCT02514551^c^ (I4T-MC-JVCZ)	II	Second-line GC	8 mg/kg Q2W 12 mg/kg Q2W	11/243	4.5
All studies				254/3216	7.9

*CAP* capecitabine, *CIS* cisplatin, *CRC* colorectal cancer, *D* day, *DOC* docetaxel, *FOLFIRI* irinotecan, folinic acid, and 5FU, *GC* gastric cancer, *HCC* hepatocellular carcinoma, *IRR* infusion-related reaction, *n *number of patients in specified category, *N *total number of patients, *NSCLC* non-small-cell lung cancer, *PAC* paclitaxel, *QW* weekly, *Q2W* every 2 weeks, *Q3W* every 3 weeks, *RAM* ramucirumab, *UC* urothelial carcinoma, *5FU* 5-fluorouracil

^a^Per protocol, all studies required RAM to be delivered over approximately 60 min (or longer, if needed to maintain an infusion rate ≤ 25 mg/min). All trial protocols required premedication to reduce the risk of infusion-related reactions

^b^Broad- and narrow-scope preferred terms were used within the Standardized Medical Dictionary for Regulatory Activities (MedDRA) queries. “Narrow” preferred terms included terms that are highly likely to represent the condition of interest and were considered sufficient to identify immediate IRRs with reasonable precision and to appropriately reflect the incidence rate of immediate IRRs

^c^Studies in combination with chemotherapy (RAINBOW: RAM + PAC; REVEL: RAM + DOC; RAISE: RAM + FOLFIRI; RAINFALL: RAM + CAP [or 5FU] + CIS; RANGE: RAM + DOC; Study JVCZ: RAM + PAC)

Any potential association between infusion rate and an increased risk of an immediate IRR was investigated using multivariate logistic regression analysis.

Statistical analyses were performed using SAS software (SAS, version 9.4). %SEE is defined as the % standard error of the estimate. It is calculated as Standard Error/Estimate × 100%**.**

## Results

### Pharmacokinetic model development and simulation result

The ramucirumab concentration–time data were well described by a two-compartment structural model parameterized in terms of CL, V_1_, peripheral volume of distribution (V_2_), and inter-compartmental CL. Drug CL was found to be time-varying and was incorporated into the model using a sigmoid function. The effect of body weight on CL and V_1_ was included and exponential interpatient variability terms were included for CL, V_1_, and V_2_. An additive inter-patient variability term was included on E_max_, with covariance between CL and V_1_, and CL and E_max_. Residual variability was accounted for by an additive/proportional error structure. Aside from body weight, none of the additional patient factors investigated for influence on the PK parameters were found to satisfy the criteria defined in a previous publication [9]. Model parameters are shown in Table S1 (online Supplementary Material).

VPC data for the 8 mg/kg Q2W and 10 mg/kg Q3W regimens are provided in Fig. S1 (online Supplementary Material). Visual examination ensured general concordance of the estimates and supported the validity of this model to describe ramucirumab concentrations in this patient population.

**Fig. 1:**
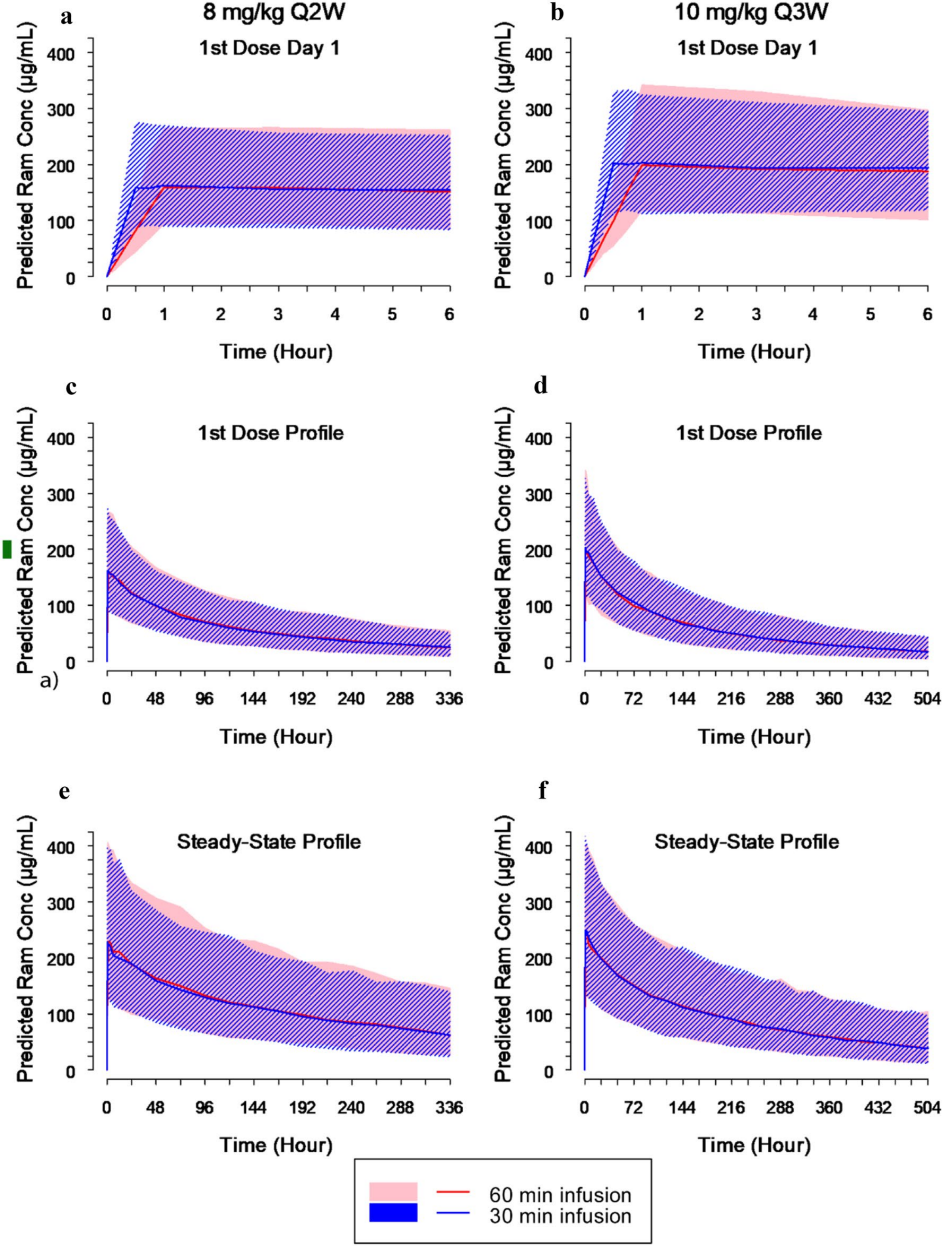
Predicted ramucirumab concentration (µg/ml) time profiles following administration of 8 mg/kg every 2 weeks (**a**, **c**, **e**) or 10 mg/kg every 3 weeks (**b**, **d**, **f**) based on a time-varying clearance population pharmacokinetic model (established using ramucirumab pharmacokinetic data from 17 studies (2522 patients). Data following the first dose (**a**–**d**) and at steady state (**e**, **f**) are shown. *CL* clearance, *conc* concentration, *Q2W* every 2 weeks, *Q3W* every 3 weeks, *Ram* ramucirumab. Shaded regions represent the 5th and 95th percentile ramucirumab concentrations calculated from 500 simulation iterations

The final PopPK model with time-varying CL was used to simulate concentration–time profiles and predict ramucirumab exposure parameters for the 8 mg/kg Q2W and 10 mg/kg Q3W regimens. Figure 1 compares the predicted concentration–time profiles following 30- and 60-min infusions. Predicted ramucirumab exposure estimates are shown in Table S3 (online Supplementary Material). These results indicate that ramucirumab PK profiles, and hence systemic exposure, are equivalent following infusions of either 30 or 60 min.

### Incidence of immediate IRRs

Table 1 summarizes the incidence of immediate IRRs in each of the ten clinical studies included in IRR analyses.

**Fig. 2:**
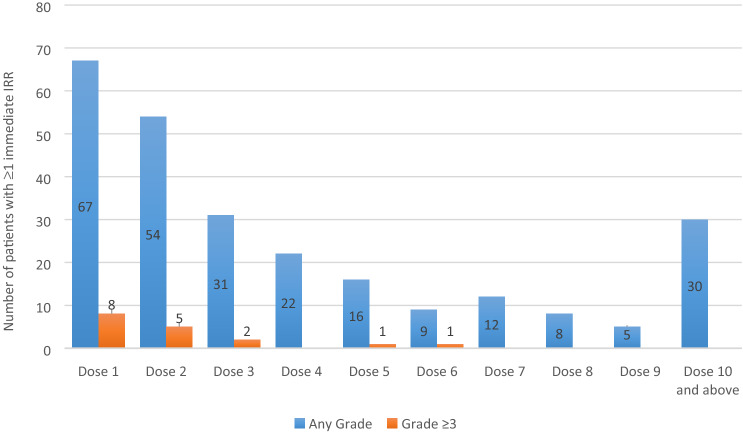
Plot of immediate infusion-related reactions (IRRs) (narrow terms^a^) by infusion number. Data shown are the number of patients with a first immediate IRR (any grade) occurring at the dose specified (blue bars), and the number of patients with a first IRR (grade ≥ 3) occurring at the dose specified (yellow bars). ^a^Broad- and narrow-scope preferred terms were used within the Standardized Medical Dictionary for Regulatory Activities (MedDRA) queries. “Narrow” preferred terms included terms that are highly likely to represent the condition of interest and were considered sufficient to identify immediate IRRs with reasonable precision and to appropriately reflect the incidence rate of immediate IRRs

In the pooled dataset, 254/3216 (7.9%) patients receiving ramucirumab experienced at least one immediate any-grade IRR (Table 1). The number of patients who experienced immediate IRRs for the first time decreased as the dose number increased (Fig. 2). Grade ≥ 3 immediate IRRs were seen in 17/3216 patients (0.5%); most occurred during the first two infusions (Fig. 2). No fatal IRRs were identified.

### Relationship between infusion rate and immediate IRR incidence

Within each study and in pooled data, the summary statistics of ramucirumab infusion rates were similar for patients with or without any immediate IRR (Table 2).

**Table 2:** Summary of infusion rates for patients with or without immediate infusion-related reactions (narrow terms^a^) by study and overall in ramucirumab-treated patients

Study			Infusion rate, mg/min	
			Pts with ≥ 1 IRR	Pts with no IRR
REGARD [21] NCT00917384		*N*	5	224
		Mean (SD)	9.8 (2.69)	8.5 (2.64)
		Median (IQR)	9.2 (8.1–12.1)	8.4 (6.9–9.6)
		Min–Max	6.6–13.1	2.4–32.5
RAINBOW^b^ [22] NCT01170663		*N*	45	279
		Mean (SD)	7.5 (1.89)	8.4 (2.15)
		Median (IQR)	7.6 (6.7–8.5)	8.0 (6.9–9.6)
		Min–Max	3.0–13.2	4.3–17.9
REVEL^b^ [23] NCT01168973		*N*	51	574
		Mean (SD)	16.2 (25.90)	12.6 (7.72)
		Median (IQR)	12.0 (9.4–13.2)	12.0 (10.3–13.9)
		Min–Max	0.5–190.0	3.5–182.0
RAISE^b^ [24] NCT01183780		*N*	45	481
		Mean (SD)	9.5 (4.84)	9.5 (2.33)
		Median (IQR)	8.8 (7.3–10.7)	9.1 (7.7–10.9)
		Min–Max	3.1–36.0	4.0–19.9
REACH [25] NCT01140347		*N*	41	275
		Mean (SD)	8.3 (2.09)	8.9 (1.95)
		Median (IQR)	8.3 (7.4–9.2)	8.5 (7.5–10.0)
		Min–Max	3.2–13.0	3.2–15.3
REACH-2 [26] NCT02435433		*N*	17	179
		Mean (SD)	7.1 (2.90)	8.6 (2.09)
		Median (IQR)	7.1 (5.7–8.4)	8.4 (7.2–9.7)
		Min–Max	1.0–11.9	3.2–17.0
RAINFALL^b^ [27] NCT02314117		*N*	15	306
		Mean (SD)	10.4 (3.04)	8.8 (2.27)
		Median (IQR)	9.7 (7.5–13.9)	8.6 (6.9–10.3)
		Min–Max	6.7–15.0	4.3–18.0
RANGE^b^ [28] NCT02426125		*N*	18	237
		Mean (SD)	11.0 (3.42)	11.9 (2.84)
		Median (IQR)	11.2 (9.6–12.9)	11.8 (10.0–13.7)
		Min–Max	3.3–16.0	5.3–23.2
NCT02443883 (I4T-MC-JVDB)		*N*	4	156
		Mean (SD)	4.8 (1.48)	9.5 (3.30)
		Median (IQR)	4.7 (3.6–6.1)	8.4 (7.1–11.4)
		Min–Max	3.4–6.4	4.0–19.5
NCT02514551^b^ (I4T-MC-JVCZ)		*N*	11	230
		Mean (SD)	11.1 (5.09)	11.1 (3.30)
		Median (IQR)	12.1 (7.3–16.0)	10.6 (8.7–13.0)
		Min–Max	2.5–18.6	4.6–25.0
All studies^c^		*N*	252	2941
		Mean (SD)	10.3 (12.35)	10.1 (4.39)
		Median (IQR)	8.7 (7.3–11.4)	9.6 (7.9–11.7)
		Min–Max	0.5–190.0	2.4–182.0

Infusion rates were calculated as total dose/duration of infusion on day of earliest IRR event for patients with at least one immediate IRR and as total dose/infusion time on the first ramucirumab dose for patients with no IRR events

Two outliers of infusion data (one with infusion rate = 182 mg/min calculated from 910 mg total dose infused within 5 min; one with infusion rate = 190 mg/min calculated from 950 mg total dose infused within 5 min; both in REVEAL) were considered due to data entry issues

*CAP* capecitabine, *CIS* cisplatin, *DOC* docetaxel, *FOLFIRI* irinotecan, folinic acid, and 5FU, *IQR* interquartile range, *IRR* infusion-related reaction, *Max* maximum, *Min* minimum, *N* total number of patients, *PAC* paclitaxel, *pts* patients, *Q* quartile, *RAM* ramucirumab, *SD* standard deviation, *5FU* 5-fluorouracil

^a^Broad- and narrow-scope preferred terms were used within the Standardized Medical Dictionary for Regulatory Activities (MedDRA) queries. “Narrow” preferred terms included terms that are highly likely to represent the condition of interest and were considered sufficient to identify immediate IRRs with reasonable precision and to appropriately reflect the incidence rate of immediate IRRs

^b^Studies in combination with chemotherapy (RAINBOW: RAM + PAC; REVEL: RAM + DOC; RAISE: RAM + FOLFIRI; RAINFALL: RAM + CAP [or 5FU] + CIS; RANGE: RAM + DOC; Study JVCZ: RAM + PAC)

^c^Infusion times were missing for 23 patients across the studies

When grouped according to infusion rate quartile, the incidence of immediate IRRs (any grade or grade ≥ 3) was similar across quartiles. There was no obvious increasing trend in IRR incidence from the lowest to the highest quartile (Table 3); similar findings were obtained in sensitivity analyses (see online Supplementary Material, including Table S4).

**Table 3:** Summary of immediate infusion-rate reactions (narrow terms^a^) by infusion rate quartile groups in ramucirumab-treated patients

		Infusion rate Q1^b^ (*N* = 799)	Infusion rate Q2^b^ (*N* = 803)	Infusion rate Q3^b^ (*N* = 797)	Infusion rate Q4^b^ (*N* = 794)	Infusion rate missing (*N* = 23)	Total (*N* = 3216)
Patients with ≥ 1 event on the day of ramucirumab administration	Any grade	87 (10.9)	65 (8.1)	40 (5.0)	60 (7.6)	2 (8.7)	254 (7.9)
	Grade ≥ 3	5 (0.6)	5 (0.6)	5 (0.6)	2 (0.3)	0	17 (0.5)
Anaphylactic reaction SMQ (narrow)	Any grade	0	1 (0.1)	1 (0.1)	1 (0.1)	0	3 (0.1)
	Grade ≥ 3	0	1 (0.1)	1 (0.1)	0	0	2 (0.1)
Angioedema SMQ (narrow)	Any grade	16 (2.0)	12 (1.5)	4 (0.5)	8 (1.0)	0	40 (1.2)
	Grade ≥ 3	0	0	0	0	0	0
Hypersensitivity SMQ (narrow)	Any grade	54 (6.8)	45 (5.6)	28 (3.5)	45 (5.7)	2 (8.7)	174 (5.4)
	Grade ≥ 3	2 (0.3)	1 (0.1)	4 (0.5)	0	0	7 (0.2)
Cytokine release syndrome (PT)	Any grade	2 (0.3)	2 (0.2)	0	0	0	4 (0.1)
	Grade ≥ 3	1 (0.1)	0	0	0	0	1 (0.0)
Infusion-related reaction (PT)	Any grade	38 (4.8)	19 (2.4)	12 (1.5)	17 (2.1)	0	86 (2.7)
	Grade ≥ 3	4 (0.5)	4 (0.5)	1 (0.1)	2 (0.3)	0	11 (0.3)

Data are presented as *n* (%)

*IRR* infusion-related reaction, *n* number of patients in specified category, *N *total number of patients, *PT* preferred term, *Q* quartile, *SMQ* Standardized Medical Dictionary for Regulatory Activities queries

^a^Broad- and narrow-scope preferred terms were used within the Standardized Medical Dictionary for Regulatory Activities (MedDRA) queries. “Narrow” preferred terms included terms that are highly likely to represent the condition of interest and were considered sufficient to identify immediate IRRs with reasonable precision and to appropriately reflect the incidence rate of immediate IRRs

^b^Range (minimum–maximum, mg/min) within each quartile group: Q1 = 0.50–7.76; Q2 = 7.76–9.50, Q3 = 9.50–11.67; Q4 = 11.67–40.29; and two outliers (182.00, 190.00)

Under multivariate logistic analysis that included infusion rate as a continuous variable together with other factors, infusion rate was not significantly associated with an increased risk of an immediate IRR (odds ratio per 1 mg/min increase 1.014; *P* = 0.071; Table 4). Identified risk factors for an immediate IRR included residing in Asia, receiving chemotherapy, and no premedication.

**Table 4:** Multivariate logistic analyses^a^ of relationships between infusion rates (continuous variable) and incidence of immediate infusion-related reactions (narrow terms^b^) in ramucirumab-treated patients

Factor	Comparison	OR (95% CI) for risk of immediate IRR	*p* value
Infusion rate	1-unit increase, mg/min	1.014 (0.999, 1.030)	0.071
Age	≥ 65 vs. < 65 years	0.831 (0.633, 1.090)	0.181
Sex	Female vs. male	1.127 (0.848, 1.497)	0.409
Region	Asia vs. rest of the world	2.151 (1.596, 2.898)	< 0.001
Premedication	Yes vs. no	0.519 (0.389, 0.691)	< 0.001
Chemotherapy	Yes vs. no	1.443 (1.033, 2.016)	0.032
Dosing regimen	10 mg/kg Q3W vs. 8 mg/kg Q2W	0.790 (0.565, 1.106)	0.170
	12 mg/kg Q2W vs. 8 mg/kg Q2W	0.456 (0.196, 1.061)	0.068
	6 mg/kg QW vs. 8 mg/kg Q2W	0.922 (0.215, 3.951)	0.913
	8 mg/kg D1D8 Q3W vs. 8 mg/kg Q2W	0.505 (0.289, 0.884)	0.017

*CI* confidence interval, *D* day, *IRR* infusion-related reaction, *OR* odds ratio, *QW* weekly, *Q2W* every 2 weeks, *Q3W* every 3 weeks

^a^Including infusion rate as a continuous variable and adjusting for other factors that might affect the incidence of immediate infusion-related reactions (age, sex, premedication, chemotherapy, region, and dosing regimen). Baseline bodyweight was not included as a covariate because of its strong correlation with infusion rate

^b^Broad- and narrow-scope preferred terms were used within the Standardized Medical Dictionary for Regulatory Activities (MedDRA) queries. “Narrow” preferred terms included terms that are highly likely to represent the condition of interest and were considered sufficient to identify immediate IRRs with reasonable precision and to appropriately reflect the incidence rate of immediate IRRs

## Discussion

Results of simulations from a PopPK model with time-varying CL established using ramucirumab PK data from 17 studies (2522 patients) found the PK profiles of ramucirumab following either a 30- or 60-min infusion to be equivalent. This finding of no change in ramucirumab exposure suggests that shortening the infusion duration of ramucirumab from 60 to 30 min would not impact efficacy or overall safety outcomes related to systemic exposure to ramucirumab.

General management strategies to reduce the risk of IRRs to mAb therapies include premedication with antihistamines and/or corticosteroids and reducing the infusion rate [11]. We investigated the perceived association between immediate IRRs and infusion rate, specifically the impact of infusion rate on the incidence of immediate IRRs. The incidence of any-grade immediate IRRs following the infusion of ramucirumab was low, with 7.9% of 3216 patients experiencing at least one immediate IRR in the ten included studies. Most immediate IRRs were grade 1 or 2; incidence of grade ≥ 3 events (requiring permanent discontinuation of ramucirumab) was low (0.5%), and most such events occurred during the first two infusions.

Although ramucirumab was administered at a range of infusion rates in the ten studies analyzed, no difference in infusion rate distributions was apparent between patients with and without at least one immediate IRR, either within each individual study or in the pooled dataset. The incidence of immediate IRRs across infusion rate quartile groups further suggested that a faster infusion rate was not associated with a higher incidence of immediate IRRs within the range of the observed infusion rate data. In fact, the highest incidence of immediate IRRs occurred in the quartile with the lowest infusion rate (Q1; 10.9% of patients had any-grade immediate IRR, 0.6% had grade ≥ 3 immediate IRR; Table 3), potentially because many of the patients who experienced an IRR would have had their infusions interrupted and infusion times prolonged (as per usual clinical practice and current labeling [4]). Sensitivity analyses, assuming a 60-min infusion for all patients, found no association between infusion rate and immediate IRR incidence. Multivariate logistic regression analyses also demonstrated that immediate IRR occurrence was not significantly associated with dose or infusion rate (mg/min) in the range of observed infusion rate data investigated. These analyses indicated that residing in Asia was associated with an increased risk of immediate IRRs. Reasons are unclear, but as we found a higher incidence of immediate IRRs in placebo-treated Asian patients than in placebo-treated non-Asian patients (data not shown) it is thought that local medical management or more stringent reporting of IRRs are more likely contributory factors to this association than ramucirumab treatment.

The mechanisms by which mAbs induce IRRs are imperfectly understood. Mechanisms proposed for rituximab (direct binding of the antibody to its antigen, resulting in cytokine release) and cetuximab (formation of IgE antidrug antibody complexes leading to allergic reactions or anaphylaxis [16, 17]) are not thought to be implicated in IRRs to ramucirumab. Retrospective and prospective studies of various therapeutic antibodies (e.g., ramucirumab, bevacizumab, rituximab, daratumumab, and infliximab), with IRR incidence as an endpoint, have found that a more rapid rate of infusion/shortened infusion time does not appear to increase IRR incidence. For example, a recent small prospective study found no immediate IRRs in patients receiving ramucirumab infused over 20 min following at least one initial 60-min infusion without evidence of immediate IRRs [18]. Hence, the commonly held perception that immediate IRR incidence is related to infusion rate warrants further investigation. Furthermore, shorter infusion times for other monoclonal antibody treatments are known to improve patient [19, 20] and clinic personal satisfaction [20] as well as lower the cost of treatment [19].

### Strengths/limitations

PopPK modeling/simulation is an established tool used during the drug development process to help predict drug concentration profiles with changing dosing regimens. The PopPK model used in these analyses was based on ramucirumab PK data from 2522 patients enrolled in 17 studies; its findings can therefore be considered robust.

The ten phase II/III clinical studies included in the evaluation of the association between ramucirumab infusion rate and the incidence of immediate IRRs enabled a thorough exploration of any potential relationship between infusion rate and immediate IRRs. Although data for higher infusion rates are sparse, IRR incidences in the literature from shorter infusion durations of therapeutic antibodies (including ramucirumab) do not seem to support a strong relationship between infusion rate and IRR incidence. We therefore consider it unlikely that increasing the infusion rate of ramucirumab (by shortening the infusion time) would impact the incidence of immediate IRRs.

This was a retrospective analysis of clinical data from historical studies and was not designed to confirm whether a shorter infusion time would affect the incidence of IRRs. The observed infusion rates that patients received might have been affected by interventions such as appropriate infusion interruptions or longer infusion times resulting from immediate IRRs, although sensitivity analyses to account for such interventions did not reveal an association. A shortened ramucirumab infusion duration could reduce overall waiting times for patients, allowing more patients to be treated, improving patient satisfaction, and lowering resource utilization. In this study, PK profiles for ramucirumab were equivalent (showing no change in drug exposure) following either a 30- or 60-min infusion. Additionally, these analyses found no evidence to suggest that decreasing the infusion time for ramucirumab would result in an increased incidence of any grade or grade ≥ 3 immediate IRRs. Given these findings, it is considered that shortening the ramucirumab infusion duration from 60 to 30 min is unlikely to impact the efficacy or safety profiles of ramucirumab. A reasonable approach would therefore be to administer the initial infusion over the currently approved 60 min and, if no IRR is observed, reduce this duration to 30 min for subsequent infusions. As of publication, the USA and Japanese regulatory authorities have updated their label to allow this dosing regimen for all approved cancer indications.

## Supplementary Information

Below is the link to the electronic supplementary material.

Supplementary file 1 (DOCX 1234 kb)
