# Supplementary material for: Evaluating clinical impact of a shortened infusion duration for ramucirumab: a model-based approach
Source: Cancer Chemother Pharmacol. 2021 Feb 2;87(5):635–45. doi: 10.1007/s00280-020-04223-9 (PMC8026424; doi:10.1007/s00280-020-04223-9)

# Supplementary material for: Evaluating clinical impact of a shortened infusion duration for ramucirumab: a model-based approach

Ling Gao^1^, Yiu-Keung Lau^1^, Ran Wei^1^, Lisa O’Brien^1^, Amanda Long^1^, Yongzhe Piao^2^, Paola Abada^1^

^1^Eli Lilly and Company, Indianapolis, USA; ^2^Eli Lilly Japan K.K., Kobe, Japan

Corresponding Author: Dr Paolo Abada, MD PhD

Address: Eli Lilly and Company
 Lilly Corporate Center

Indianapolis,

IN 46285

U.S.A.

Phone: +1 317 294 4583

Email: [abada_paolo@lilly.com](mailto:abada_paolo@lilly.com)

Funding: This work was supported by Eli Lilly and Company.

Table S1. Pharmacokinetic parameters from the final population model for ramucirumab (established using pharmacokinetic data for ramucirumab from 17 studies [2522 patients]). Summary statistics of ramucirumab were similar for patients with or without immediate IRR

| **Parameter description** | **Population estimate  (%SEE)** | **Interpatient variability (%SEE)** |
| --- | --- | --- |
| Clearance |  |  |
| Parameter for CL (L/hr) | 0.0205 (3.23) | 36.6% (7.92) |
| Effect of body weight on CL^a^ | 0.487 (6.49) | --- |
| Central volume of distribution |  |  |
| Parameter for V_1_ (L) | 3.31 (0.671) | 23.0% (6.82) |
| Effect of body weight on V_1_^b^ | 0.572 (4.93) | --- |
| Inter-compartmental clearance (L/hr) | 0.0269 (12.9) | --- |
| Peripheral volume of distribution; V_2_ (L) | 1.23 (8.70) | 88.5% (22.7) |
| Maximum change in CL; E_max_ | -0.418 (12.2) | 67.6% (21.4) |
| Time to half-maximal change in CL; T_50_ (hr) | 647 (10.4) | --- |
| Hill coefficient | 1.67 (18.5) | --- |
| Interpatient variability correlation coefficient |  | |
| CL and V_1_ | 0.567 (6.72) | |
| CL and T_max_ | -0.474 (25.2) | |
| Residual error |  | |
| Additive (µg/mL) | 2.34 (12.5) | |
| Proportional | 21.1% (6.03) | |

CL, clearance; SEE, standard error of the estimate; T_50_, time to half-maximal change in CL; E_max_, maximum change in clearance; V_1_, central volume of distribution; V_2_, peripheral volume of distribution.

^a^CL = 0.0205 × (body weight/68)^0.487^, where 68 is the median baseline body weight.

^b^V_1_ = 3.31 × (body weight/68)^0.572^, where 68 is the median baseline body weight.

ETA shrinkage (%): CL: 17.8, V1: 24.2, V2: 57.6, T_max_ 51.3; EPS shrinkage (%): 18.5.

## *Identification and calculation of immediate infusion-related reactions (IRRs), and investigation of the relationship between immediate IRR incidence and infusion rate*

Clinical data from ten clinical studies that evaluated ramucirumab (8–12 mg/kg) were included in analyses to evaluate the association between ramucirumab infusion rate and the incidence of immediate IRR events. Immediate IRRs (events occurring on the same day as ramucirumab infusion) were identified from Standardized Medical Dictionary for Regulatory Activities (MedDRA) queries (SMQs) and preferred term (PT) search criteria (anaphylactic reaction SMQ, hypersensitivity SMQ, angioedema SMQ, PT infusion-related reaction, and PT cytokine‑release syndrome).

Broad- and narrow‑scope PTs were used within the three SMQs. MedDRA SMQ “narrow” PTs included terms that are highly likely to represent the condition of interest; “broad” PTs included additional terms that may represent the condition of interest. Following physician review of each event identified using narrow or broad PTs, the identification of immediate IRRs using SMQ narrow PTs was considered sufficient to identify immediate IRRs with reasonable precision and to appropriately reflect the incidence rate of immediate IRRs.

The number (%) of patients with at least one immediate IRR was calculated by study and across all studies. Additionally, the infusion rate (mg/min) for each patient was calculated as the total dose administered divided by the infusion duration of the first ramucirumab dose for patients with no immediate IRR events, or on the day of the earliest IRR event for patients with at least one defined immediate IRR events.

Pooled data for all ramucirumab-treated patients were categorized into groups based on the quartiles of their infusion rates calculated either on the day of the first ramucirumab dose (if no IRR occurred) or on the day of the earliest IRR event. The incidence of immediate IRR events was summarized within each quartile group to investigate a potential trend of increased IRR incidence from lower quartile to higher quartile groups.

Sensitivity analysis investigated the association between immediate IRR incidence and the infusion rate that patients were supposed to receive over a standard 60-minute infusion based on dose level and body weight (calculated as: planned dose level × body weight/60).

Table S2. Studies included in the updated population pharmacokinetic model (including data from a total of 2522 patients)

| **Study** | **Indication** | **Ramucirumab dosing regimen** | **Patients included in pharmacokinetic assessment (n)** |
| --- | --- | --- | --- |
| REACH-2 (I4T-MC-JVDE) | Hepatocellular carcinoma | 8 mg/kg Q2W | 193 |
| I4T-CR-JVCQ | Hepatocellular carcinoma | 8 mg/kg Q2W | 8 |
| I4Y-IE-JCDC | Urothelial carcinoma | 10 mg/kg Q3W | 39 |
| RANGE (I4T-MC-JVDC)a | Urothelial carcinoma | 10 mg/kg Q3W | 239 |
| I4T-MC-JVDB | Gastric or gastroesophageal junction adenocarcinoma | 6 mg/kg QW  8mg/kg Q2W  8 mg/kg D1D8 Q3W  12 mg/kg Q2W | 161 |
| RAISE (I4T-MC-JVBB) | Colorectal carcinoma | 8 mg/kg Q2W | 431 |
| RAINBOW (I4T-IE-JVBE) | Gastric or gastroesophageal junction adenocarcinoma | 8 mg/kg Q2W | 321 |
| REACH (I4T-IE-JVBF) | Hepatocellular carcinoma | 8 mg/kg Q2W | 312 |
| REVEL (I4T-MC-JVBA) | Non-small-cell lung cancer | 10 mg/kg Q3W | 399 |
| REGARD (I4T-IE-JVBD) | Gastric cancer or gastroesophageal junction adenocarcinoma | 8 mg/kg Q2W | 72 |
| I4T-IE-JVBJ | Non-small-cell lung cancer | 10 mg/kg Q3W | 32 |
| I4T-IE-JVBW | Gastric or gastroesophageal junction adenocarcinoma | 8 mg/kg Q2W | 6 |
| I4T-IE-JVBX | Breast cancer | 10 mg/kg Q3W | 7 |
| I4T-IE-JVBY | Colorectal carcinoma | 8 mg/kg Q2W | 6 |
| I4T-IE-JVCA | Solid tumors | 8 mg/kg Q2W | 36 |
| I4T-IE-JVCC | Solid tumors | 10 mg/kg Q3W | 17 |
| I4T-MC-JVCZ | Gastric cancer or gastroesophageal junction adenocarcinoma | 8 mg/kg Q2W  12 mg/kg Q2W | 243 |

D, day; QW, weekly; Q2W, every 2 weeks; Q3W, every 3 weeks

Table S3. Population pharmacokinetic modeling and simulation-derived exposure estimates

|  | | Ramucirumab exposure estimate (μg/mL) | |
| --- | --- | --- | --- |
| Dosing regimen and infusion duration | Parameter^a^ | Typical value for mean (minimum–maximum)^b^ weight in simulation dataset | Population prediction  median (90%PI) |
| 8 mg/kg Q2W | | | |
| 60 min | C_min1_ | 27.3 (19.8–38.3) | 23.9 (8.72–54.4) |
|  | C_max1_ | 165 (125–221) | 159 (93.0–265) |
|  | C_min,ss_ | 65.4 (48.2–91.1) | 61.6 (27.9–146) |
|  | C_max,ss_ | 230 (172–310) | 229 (132–406) |
| 30 min | C_min1_ | 27.2 (19.7–38.3) | 25.2 (8.80–52.0) |
|  | C_max1_ | 166 (125–221) | 158 (88.0–276) |
|  | C_min,ss_ | 65.4 (48.1–91.0) | 62.1 (23.4–140) |
|  | C_max,ss_ | 230 (173–311) | 229 (116–402) |
| 10 mg/kg Q3W | | | |
| 60 min | C_min1_ | 18.3 (13.8–24.9) | 17.4 (4.05–42.4) |
|  | C_max1_ | 207 (156–276) | 199 (117–342) |
|  | C_min,ss_ | 40.3 (30.4–55.3) | 38.5 (13.3–104) |
|  | C_max,ss_ | 246 (186–330) | 243 (129–402) |
| 30 min | C_min1_ | 18.2 (13.8–24.9) | 16.7 (4.45–43.9) |
|  | C_max1_ | 207 (157–277) | 202 (113–330) |
|  | C_min,ss_ | 40.3 (30.3–55.3) | 38.1 (11.3–97.2) |
|  | C_max,ss_ | 246 (186–330) | 241 (139–423) |

min, minutes; PI, prediction interval; Q2W, every 2 weeks; Q3W, every 3 weeks.

^a^C_min1_ is the minimum concentration following the first dose (336 h for Q2W and 504 h for Q3W); C_min,ss_ is the minimum concentration at the steady state (week 18 [3024 h] following nine doses for Q2W and six doses for Q3W); C_max1_ is the concentration at the end of infusion following the first dose; C_max,ss_ is the concentration at the end of infusion at the steady state (following nine doses for Q2W and six doses for Q3W).

^b^For 500 simulation patients, mean = 70 kg, minimum = 37 kg, and maximum = 137 kg.

Table S4. Summary of immediate infusion-rate reactions (narrow terms^a^) by quartile groups on infusion rate during a standard 60-minute infusion in ramucirumab-treated patients (sensitivity analysis)

|  | | **Infusion rate Q1^b^ (N=807)** | **Infusion**  **rate Q2^b^ (N=802)** | **Infusion**  **rate Q3^b^ (N=804)** | **Infusion**  **rate Q4^b^ (N=803)** | **Total (N=3216)** |
| --- | --- | --- | --- | --- | --- | --- |
| Patients with ≥1 event on the day of ramucirumab administration | Any  grade | 66 (8.2) | 71 (8.9) | 45 (5.6) | 72 (9.0) | 254 (7.9) |
|  | Grade  ≥3 | 4 (0.5) | 4 (0.5) | 7 (0.9) | 2 (0.2) | 17 (0.5) |
| Anaphylactic reaction SMQ | Any grade | 0 | 1 (0.1) | 1 (0.1) | 1 (0.1) | 3 (0.1) |
|  | Grade  ≥3 | 0 | 1 (0.1) | 1 (0.1) | 0 | 2 (0.1) |
| Angioedema SMQ | Any grade | 12 (1.5) | 14 (1.7) | 6 (0.7) | 8 (1.0) | 40 (1.2) |
|  | Grade ≥3 | 0 | 0 | 0 | 0 | 0 |
| Hypersensitivity SMQ | Any grade | 41 (5.1) | 58 (7.2) | 26 (3.2) | 49 (6.1) | 174 (5.4) |
|  | Grade ≥3 | 1 (0.1) | 2 (0.2) | 4 (0.5) | 0 | 7 (0.2) |
| Cytokine release syndrome (PT) | Any grade | 2 (0.2) | 1 (0.1) | 1 (0.1) | 0 | 4 (0.1) |
|  | Grade ≥3 | 0 | 1 (0.1) | 0 | 0 | 1 (0.0) |
| Infusion‑related reaction (PT) | Any grade | 27 (3.3) | 16 (2.0) | 18 (2.2) | 25 (3.1) | 86 (2.7) |
|  | Grade ≥3 | 3 (0.4) | 3 (0.4) | 3 (0.4) | 2 (0.2) | 11 (0.3) |

Data are presented as n (%)

IRR, infusion-related reaction; n, number of patients in specified category; N, total number of patients; PT, preferred term; Q, quartile; SMQ, Standardized Medical Dictionary for Regulatory Activities queries.

^a^Broad- and narrow-scope preferred terms were used within the Standardized Medical Dictionary for Regulatory Activities (MedDRA) queries. “Narrow” preferred terms included terms that are highly likely to represent the condition of interest and were considered sufficient to identify immediate IRRs with reasonable precision and to appropriately reflect the incidence rate of immediate IRRs.

^b^Range (min–max, mg/min) within each quartile: Q1=3.02–8.12; Q2=8.12–9.76; Q3=9.76–11.88; Q4=11.88–25.00.

Fig S1. Visual predictive check of the final pharmacokinetic model for the ramucirumab 8 mg/kg every 2 weeks (Q2W; left panels) and 10 mg/kg every 3 weeks (Q3W; right panels) regimens. Top panels (a,b) depict data collected within the first 647 hours (27 days) of treatment. Bottom panels (c,d) depict data collected ≥647 hours (27 days) after treatment initiation. Dashed lines represent the 5th, 50th, and 95th percentiles of the observed concentration data. Solid lines represent the 5th, 50th, and 95th percentiles of model predictions


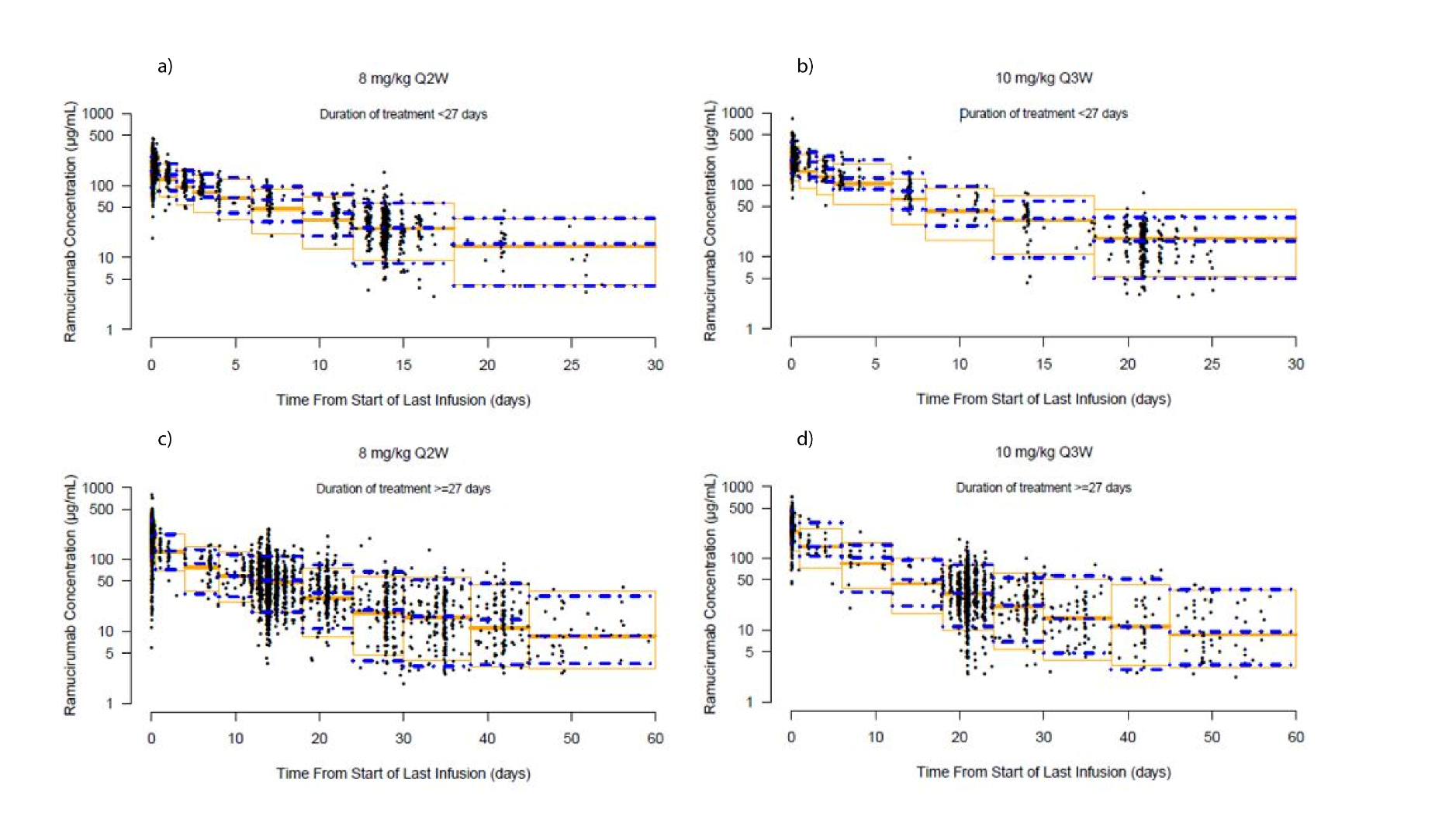

Supplement: Supplementary file 1 — (DOCX 1234 kb) [file 280_2020_4223_MOESM1_ESM.docx]
